# TRIM22: A Diverse and Dynamic Antiviral Protein

**DOI:** 10.1155/2012/153415

**Published:** 2012-05-08

**Authors:** Clayton J. Hattlmann, Jenna N. Kelly, Stephen D. Barr

**Affiliations:** Department of Microbiology and Immunology, Center for Human Immunology, The University of Western Ontario, London, ON, Canada N6A 5C1

## Abstract

The tripartite motif (TRIM) family of proteins is an evolutionarily ancient group of proteins with homologues identified in both invertebrate and vertebrate species. Human TRIM22 is one such protein that has a dynamic evolutionary history that includes gene expansion, gene loss, and strong signatures of positive selection. To date, TRIM22 has been shown to restrict the replication of a number of viruses, including encephalomyocarditis virus (EMCV), hepatitis B virus (HBV), and human immunodeficiency virus type 1 (HIV-1). In addition, TRIM22 has also been implicated in cellular differentiation and proliferation and may play a role in certain cancers and autoimmune diseases. This comprehensive paper summarizes our current understanding of TRIM22 structure and function.

## 1. Introduction

The TRIM gene family encodes a diverse group of proteins that are involved in many biological and antiviral processes. There are currently 100 known TRIM genes in the human genome and many of these genes are upregulated by multiple, distinct stimuli [1–3]. Historically, TRIM genes have been researched mainly for their antiviral properties; however this paradigm is changing. Two recent reports discussing the role of TRIM genes in autoimmunity and cancer highlight the importance of the TRIM family in the development of nonviral diseases [4, 5]. Many TRIM genes also have a dynamic evolutionary history and the TRIM family has been shown to undergo extensive gene duplication in both primates and teleost fish [1, 6]. In addition, several TRIM genes have experienced strong positive selection in primates [7]. Although the forces behind TRIM evolution remain unclear, it is possible that the TRIM family has evolved and continues to evolve, in response to new viral pathogens or endogenous danger signals. This paper provides an overview of the TRIM22 gene and summarizes its structure, evolution, expression, and antiviral activities.

## 2. Structure

**Figure 1:**
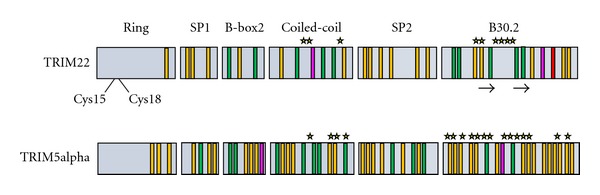
Structure and variability of TRIM22 and TRIM5*α* protein domains. TRIM22 contains an amino-terminal RING domain, one B-box domain (B-box 2), a coiled-coil region, and a carboxyl-terminal B30.2 domain (SP1 = Spacer 1 and SP2 = Spacer 2). Two cysteine residues (Cys15 and Cys18) in the RING domain are required for the E3 ligase activity of TRIM22, and a number of positively selected amino acids are found in the coiled-coil and B30.2 domains. The location and spacing of positively selected amino acids in TRIM22 are similar to those found in TRIM5*α*, which may reflect species-specific pathogenic pressures. The approximate location of positively selected amino acids in TRIM22 and TRIM5*α* is denoted with a star, and the location of the *β*2-*β*3 surface loop of TRIM22 is also indicated (arrows). Single nucleotide polymorphisms (SNPs) in the coding regions of TRIM22 and TRIM5*α* are shown as vertical bars, along with the type of mutation that each SNP can generate (green: nonsynonymous mutations; yellow: missense mutations; pink: frameshift mutations; red: nonsense mutations).

TRIM proteins typically contain a conserved RBCC motif, which consists of an amino-terminal RING domain, one or two B-box domains, and a predicted coiled-coil region. Approximately 60% of TRIM proteins, including TRIM22, also contain a carboxyl-terminal domain B30.2 domain (Figure 1) [8, 9]. The RING domain of TRIM22 has homology with E3 ligases and has been shown to possess E3 ubiquitin ligase activity [9, 10]. The catalytic cysteine residues Cys15 and Cys18 are essential for this activity and mediate the transfer of ubiquitin to target proteins (Figure 1) [11, 12]. TRIM22 can also modify itself with ubiquitin which leads to proteasomal degradation [10, 11]. Interestingly, the *TRIM* family represents one of the largest groups of E3 ubiquitin ligases and E3 ligase activity seems to be crucial for *TRIM*-mediated carcinogenesis [4]. In addition, E3 ligase activity is important for many *TRIM*-mediated antiviral activities and for TRIM22, it is required for the inhibition of EMCV, HBV, and HIV-1 [11, 13, 14].

TRIM proteins typically contain one or two B-box domains, although B-box 1 is never present without B-box 2, and the two domains have different consensus sequences [15, 16]. TRIM22 contains one B-box domain (B-box 2), of which no clear function has been assigned (Figure 1). Certain B-box 2 mutations have been shown to affect viral recognition by other TRIM proteins, such as TRIM5*α*. Similar to TRIM22, TRIM5*α* has been shown to inhibit HIV-1 replication albeit at an earlier stage in the viral lifecycle. Interestingly, the human orthologue of TRIM5*α* only modestly inhibits HIV-1 replication whereas the rhesus orthologue of TRIM5*α* (rhTRIM5*α*) has potent anti-HIV-1 activity [17]. Several mechanisms of rhTRIM5*α*-mediated HIV-1 inhibition have been proposed; however, the favoured mechanism involves rhTRIM5*α* binding to the HIV-1 core and disruption of the normal uncoating process (reviewed in [18, 19]). For rhTRIM5*α*, the RING and B-box 2 domains promote its dimerization and higher-order self-association on the HIV-1 capsid [17]. It is unknown whether the B-box 2 domain of TRIM22 is required for higher-order self-association; however, it has been shown to play a role in the nuclear localization of TRIM22 [20].

The coiled-coil domain contains multiple predicted hypersecondary structures and intertwined *α*-helices [21]. In TRIM proteins, the coiled-coil domain is thought to promote homo-oligomerization, as its deletion prevents TRIM protein self-association [22]. Homo-oligomerization can be important for the formation of higher-molecular-weight complexes that define specific subcellular structures, such as nuclear bodies [21, 22]. Although the role of the coiled-coil region of TRIM22 remains unclear, self-association is a function of the coiled-coil region in other TRIM proteins. For example, the coiled-coil region of rhTRIM5*α* is required for rhTRIM5*α* trimerization and may be involved in the formation of cytoplasmic bodies. Importantly, rhTRIM5*α* trimerization is thought to drive its interaction with the HIV-1 capsid and the coiled-coil region is required for rhTRIM5*α*-mediated HIV-1 restriction [17, 23]. TRIM22 has also been shown to form trimers and to restrict HIV-1 replication but it is unknown whether the coiled-coil domain is required for these processes [14, 22, 24–26].

The B30.2 domain of TRIM proteins consists of two separate domains called the PRY and SPRY domains that form a putative protein-protein interaction site [27, 28]. This interaction site is likely important for the antiviral activities of TRIM22 and other TRIM proteins. Indeed, the B30.2 domain of rhTRIM5*α* is required for trimerization and HIV-1 restriction [23]. Three hyper-variable regions in the B30.2 domain of rhTRIM5*α* are thought to form the binding surface for the HIV-1 capsid protein [29]. In addition, these hypervariable regions confer the virus specificity of rhTRIM5*α*. The B30.2 domain of TRIM22 also contains these three hypervariable regions but their role in HIV-1 restriction has not yet been established. Similar to rhTRIM5*α*, the hypervariable regions in TRIM22 are highly polymorphic and contain a large number of positively selected amino acids (Figure 1) [7]. It will be interesting to learn if the B30.2 domain of TRIM22 confers specificity for targets such as viral pathogens. Notably, the B30.2 domain is required for the formation of nuclear bodies [13, 20, 30].

## 3. Evolution of TRIM22

**Figure 2:**
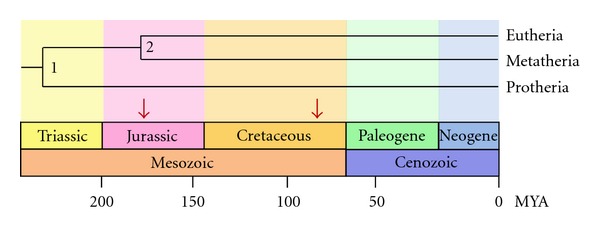
Timeline of Metatherian and Eutherian mammalian evolution showing the emergence of *TRIM22*. The divergence of Metatherian (marsupial) and Eutherian (placental) mammals occurred approximately 180 million years ago in the Jurassic period of the Mesozoic era. The *TRIM22* gene emerged sometime after this division, as it is absent in Metatherian mammals but present in all major Eutherian groups. In addition, since *TRIM22* is present in all Eutherian mammals, it must have emerged before further Eutherian division occurred (approximately 90 million years ago). Taken together, this dates the birth of *TRIM22* to approximately 90–180 million years ago. The predicted window of time for *TRIM22* emergence in Eutherian mammals is demarcated with two red arrows. MYA: millions of years.

The human *TRIM22* gene is located on chromosome 11, immediately adjacent to the *TRIM5*, *TRIM6, *and *TRIM34 *genes [7, 31]. The origins of *TRIM22,* and the entire *TRIM5/6/22/34* gene cluster, can be traced back to the Cretaceous period, sometime after the divergence of Metatherian (marsupial) and Eutherian (placental) mammals (Figure 2). Previous studies have shown that the *TRIM5/6/22/34* locus is absent in Metatherian mammals such as opossum and chicken but presents in the major Eutherian groups containing cow, dog, and human [7]. Thus, this gene cluster must have emerged after the Metatherian-Eutherian division but before the separation of the major Eutherian groups. Taken together, this dates the birth of *TRIM22* (along with *TRIM5, TRIM6 and TRIM34*) to approximately 90–180 million years ago (Figure 2) [7].

The *TRIM5/6/22/34* gene cluster likely arose through tandem gene duplication, as these four *TRIM* genes are close human paralogs and because major gene rearrangements have been documented in this chromosomal region [1, 7, 32]. Gene duplication plays a major role in evolution and *TRIM* genes have been shown to undergo extensive gene duplication in both primates and teleost fish [1, 6]. One of the most important outcomes of gene duplication is neofunctionalization, whereby one copy of the duplicate gene acquires a novel, beneficial function, and the other copy of the gene retains its original function [33–35]. This type of gene manipulation is a potent driver of evolution because it allows an organism to create new, potentially advantageous genes without disrupting the integrity of the original gene.

Recently, a genomic analysis of a different branch of the *TRIM* gene family identified several *TRIM* genes on chromosome 11 that have given rise to multiple *TRIM* paralogs in humans and African apes [1]. A group of 7 *TRIM* genes that are present in all Eutherian mammals (*TRIM43*, *TRIM48*, *TRIM49*, *TRIM51*, *TRIM53*, *TRIM64,* and *TRIM77*) were shown to spawn 11 new *TRIM* genes in certain primates and 6 new *TRIM* genes in humans, primarily through segmental duplications [1]. These new *TRIM* genes have presumably evolved and adapted to react against more recently emerged pathogenic threats. In addition, a Han Chinese woman with 12 new *TRIM* genes was identified, documenting for the first time *TRIM* gene copy number variation in humans [1]. Given its role in antiviral immunity, *TRIM22* probably emerged in a similar manner as a means of counteracting new viral pathogens; however the exact selective pressures giving rise to the *TRIM22 *gene remain unclear.

According to a recent study, *TRIM* genes can be divided into two main groups based on their structural similarities and evolutionary properties [36]. Group 1 members have two B-box domains, have variable C-terminal domains, and are represented in both vertebrate and invertebrate species. In contrast, Group 2 members have only one B-box domain (B-box 2), are characterized by a C-terminal SPRY domain, and are found only in vertebrates. In addition, Group 2 genes are younger and smaller and evolve more rapidly than Group 1 genes [36]. Compared to some other *TRIM *genes, *TRIM22 *is young and has evolved under strong positive selection, thus *TRIM22 *(along with the *TRIM5/6/22/34* gene cluster) is classified as a Group 2 gene. Interestingly, the authors suggest that Group 2 genes may act as *TRIM* gene reservoirs, spawning new genes to respond to species-specific changes at the host-pathogen interface. Consistent with this interpretation, there are a number of positively selected amino acids in TRIM22 which all cluster at predicted virus interaction sites in the coiled-coil and B30.2 domains (Figure 1) [7, 36].

Within the *TRIM5/6/22/34* gene cluster, *TRIM22* and *TRIM5* have a unique evolutionary relationship. In some Eutherian groups, such as cow, there are multiple copies of the *TRIM5* gene and no *TRIM22* gene. However in others such as dog, the *TRIM22* gene is present and the *TRIM5* gene is absent [7]. In addition, the strong positive selection that each of these two genes has experienced over millions of years has occurred in a mutually exclusive manner. This type of anticorrelative pattern is probably due to genetic linkage between the two genes, whereby positive selection of an advantageous mutation in one gene indirectly leads to the selection of a linked mutation in the other [7]. The location and spacing of positively selected amino acids in TRIM22 is very similar to those found in TRIM5*α* (Figure 1). In both proteins, the positively selected amino acids are located in the coiled-coil and B30.2 domains, which is interesting because their amino acid sequences are actually the least similar in these regions. The majority of positively selected amino acids in TRIM22 are found within the *β*2-*β*3 surface loop of the B30.2 domain, an area that is important for HIV-1 recognition in TRIM5*α* (Figure 1) [7, 37, 38]. It is possible that TRIM22 and TRIM5*α* once possessed a similar antiretroviral mechanism, and that they evolved over time to respond to species-specific pathogenic pressures. Indeed, many studies have shown that rhesus TRIM5*α*, but not human TRIM5*α*, can potently inhibit HIV-1 replication [18, 39]. In contrast, human TRIM22 can inhibit HIV-1 replication and thus may have evolved to compensate for the loss of TRIM5*α*'s anti-HIV function.

The *TRIM22* gene has a dynamic evolutionary history that includes gene expansion, gene loss, and strong signatures of positive selection in primates [1, 6, 7, 36]. The high number of nonsynonymous mutations found in *TRIM22*, along with its classification as a Group 2 *TRIM* gene, suggests that this gene continues to evolve at a rapid pace. Given the volatile state of other *TRIM *genes in chromosome 11, it is possible that the *TRIM5/6/22/34* gene cluster takes part in gene and/or segmental duplication in humans. Presumably, individuals with an increased number of these *TRIM* genes may have an augmented antiviral response and could be particularly adept at controlling retroviral infections. Similar to copy number variations, a number of single nucleotide polymorphisms (SNPs) exist in *TRIM22* that may influence its antiviral capacity or biological function for that matter. For instance, there are two documented frameshift mutations and one documented nonsense mutation in the National Center for Biotechnology Information SNP database for the *TRIM22 *gene (Figure 1). If present, these SNPs would generate different truncated versions of the TRIM22 protein, which may alter its structure, E3 ubiquitin ligase activity and/or antiviral function. There are also twenty documented missense mutations in the *TRIM22* gene, the majority of which are found in its B30.2 domain (Figure 1). Many of these SNPs have the potential to impact TRIM22 function and their presence or absence may contribute to individual differences in TRIM22-mediated activities.

## 4. Biological Functions of TRIM22

### 4.1. TRIM22 Localization

There are several contradictory reports detailing the subcellular localization of TRIM22. Some reports have observed that TRIM22 localizes predominantly to the cytoplasm [22, 40] or to the nucleus [10, 13, 20, 41], whereas other reports have observed that TRIM22 can localize to both the cytoplasm and the nucleus (Table 1) [30, 42, 43]. The pattern of localization also varied between diffuse, speckled, and aggregated. A number of explanations have been given in the literature for the differences in localization, including whether the expression was endogenous (e.g., IFN-treatment) or exogenous (e.g., overexpression). In addition, the method of fixation and the type of epitope tag used for detection have also been reported to affect the localization pattern. Given the diverse range of cell lines used in these studies, it is also possible that cell type-specific factors influence the localization of TRIM22.

A number of determinants affecting TRIM22 localization have been identified. A bipartite nuclear localization signal (NLS) located in the Spacer 2 domain of TRIM22 was shown to be necessary, but not sufficient, for nuclear localization [20]. Although there are no known NLSs present in the B30.2/SPRY domain, several groups have shown that this domain is required for nuclear localization [13, 20, 40, 41]. More specifically, Val 493 and Cys 494 of the B30.2 domain were shown to be critical for nuclear localization and the formation of nuclear bodies [20]. In an independent study, amino acids Ser 395, Lys 396, and Ser 400 located in variable loops 1 and 3 of the B30.2 domain were shown to be important for certain localization patterns of TRIM22 [40].

In some cell types, TRIM22 localizes in the nucleus as punctate bodies, which have been shown to partially colocalize with Cajal bodies [20]. Cajal bodies play important roles in RNA processing and modification as well as in cell cycle progression [44]. TRIM22 also interacts with p80-coilin, which is a major component of Cajal bodies. Similar to Cajal bodies, TRIM22 localization has been shown to change during the cell cycle. In G0/G1 TRIM22 localizes in nuclear bodies, in S-phase it localizes in a more diffuse and speckled pattern throughout the nucleus, and during mitosis it assumes a diffuse pattern in both the nucleus and cytoplasm [30]. In an independent study, TRIM22 was shown to colocalize with the centrosome independently of the cell cycle and also with vimentin-containing aggresome-like structures next to the endoplasmic reticulum [42]. From these data, it appears that multiple factors influence the localization of TRIM22, possibly indicating that TRIM22 has several biological roles.

### 4.2. Antiviral Function of TRIM22

Several reports including published transcriptional profiling datasets (e.g., GDS1096, GDS3113, and GDS596) deposited in the Gene Expression Omnibus database repository (http://www.ncbi.nlm.nih.gov/gds) show that TRIM22 is ubiquitously expressed in several human tissues and is highly upregulated in response to Type I and II interferons (Table 2) [7, 13, 14, 24, 25, 45–50]. Interestingly, the 5′ flanking region of the *TRIM22* gene contains two regions matching the consensus sequence for an IFN-stimulating response element (ISRE) and a third region matching that for an IFN-*γ* activation site (GAS); however ISRE1 or GAS is not required for IFN-*γ* induction of TRIM22. In contrast, the ISRE2 plus six upstream nucleotides (extended ISRE) is capable of binding IFN regulatory factor 1 (IRF1) in a manner dependent on the chromatin remodelling enzyme Brahma-related gene 1 (BRG1) [48, 49]. Furthermore, this extended ISRE appears to be important for both stimulation by IFN-*α* and IFN-*γ* as well as for basal TRIM22 expression [48]. The significant upregulation of TRIM22 in response to IFNs, together with the finding that TRIM22 has evolved under strong positive selection for millions of years, suggests that TRIM22 plays an important fundamental role in cell biology. To date, several lines of evidence suggest that this role is as an antiviral factor.

Human TRIM22 was first discovered by Tissot and Mechti in 1995 during a search for IFN-induced genes in Daudi cells, where exogenous expression of TRIM22 was shown to downregulate transcription from the HIV-1 LTR [45]. Although this was performed using a luciferase reporter gene under the transcriptional control of the HIV-1 LTR and not in the context of the entire HIV-1 proviral genome, it provided the first evidence suggesting that TRIM22 blocks HIV-1 transcription and replication. In 2006, Bouazzaoui et al. showed that TRIM22 was highly upregulated in primary monocyte-derived macrophages (MDMs) in response to HIV-1 infection, IFN*α* treatment, or stimulation with lipopolysaccharide (LPS). They provided the first evidence that TRIM22 can restrict HIV-1 replication *in vitro* by showing that exogenous expression of TRIM22 inhibited HIV-1 infection by 50–90% in 293T cells modified to express the CD4 and CCR5 receptors and in primary MDM. Furthermore, cotransfection of TRIM22 with a three-plasmid system for replication-defective HIV-1 resulted in reduced infectious titres of pseudotyped virus, suggesting that TRIM22 inhibited a late stage of HIV-1 pseudoparticle production and/or subsequent infection with the pseudotyped virus [24].

**Figure 3:**
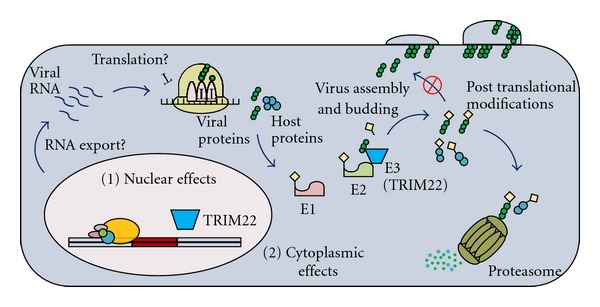
Possible mechanisms of TRIM22 antiviral functions. Based on current reports, TRIM22 can inhibit viral replication through nuclear-associated effects such as inhibiting viral transcription. Although not investigated to date, RNA export and translation are also potential targets of TRIM22. Given its E3 ligase activity, TRIM22 may posttranslationally modify host or viral proteins that are required for viral assembly and/or budding. Posttranslational modifications occur when an E1 activating enzyme (E1), E2 conjugating enzyme (E2), and E3 ligase protein (E3) work together to transfer ubiquitin or ubiquitin-like molecules to a target protein. These modifications could target the protein for proteasomal degradation or alter its subcellular localization or ability to interact with other proteins or DNA.

In 2008, Barr et al. showed that TRIM22 was an integral part of the Type I interferon-induced inhibition of HIV-1 replication and provided the first mechanistic data for the inhibition of HIV-1 replication by TRIM22. TRIM22 expression in several human cell lines potently inhibited HIV-1 replication, and interestingly, analysis of Gag production in those cells revealed that TRIM22 may inhibit HIV-1 replication by two different mechanisms. In the HOS and HeLa cell lines, TRIM22 inhibited HIV-1 particle production by interfering with the trafficking of the Gag polyprotein to the plasma membrane. Since TRIM22 and Gag proteins interact, and that the E3 ligase activity of TRIM22 is required for this restriction [14], it is possible that TRIM22 posttranslationally modifies Gag, resulting in altered Gag trafficking to the plasma membrane. In the U2OS and 143B cell lines, TRIM22 inhibited HIV-1 particle production by inhibiting the accumulation of intracellular Gag protein [14]. Although no mechanism of restriction was identified in U2OS or 143B cells, several possibilities could explain the decrease in intracellular Gag protein levels, including inhibition of transcription from the LTR as previously suggested [25, 45], or degradation of the Gag RNA and/or polyprotein. Given that TRIM22 exhibits cell type-specific differences in localization (as discussed earlier), it is likely that the mechanism of TRIM22-induced restriction of HIV-1 particle production is cell type-specific and/or dependent on the subcellular localization of TRIM22. Future experiments are required to further elucidate the mechanism of TRIM22-induced inhibition of HIV-1 particle production (Figure 3).

TRIM22 was also independently identified and shown to inhibit HIV-1 replication by several laboratories [25]. Following observations made by Franzoso et al. in 1994 that clones of the U937 promonocytic cell line were either permissive or nonpermissive to HIV-1 replication, Kajaste-Rudnitski et al. (2011) identified *TRIM22 *as the only known restriction factor that was expressed in the nonpermissive and absent from the permissive U937 cells. Using a luciferase reporter plasmid under the control of the HIV-1 LTR, they showed that LTR-mediated transcription was decreased 7–10-fold in nonpermissive clones. They also showed that by knocking down *TRIM22* expression in nonpermissive cells, the levels of transcription from the LTR approached those observed in permissive cells. Exogenous expression of TRIM22 in permissive clones also decreased LTR transcription to levels comparable to those observed in nonpermissive clones. Further investigation revealed that TRIM22 inhibited basal and phorbol myristate acetate-ionomycin-induced HIV-1 transcription. These effects were independent of NF*κ*B, HIV-1 Tat and the E3 ubiquitin ligase activity of TRIM22 [25]. It is important to note that all direct evidence showing that TRIM22 inhibits HIV-1 transcription has been through the use of LTR-driven reporter constructs. It will be important to test the effects of TRIM22 on HIV-1 LTR transcription in the context of full-length replication-competent HIV-1.

In 2011, Singh et al. provided the first clinically relevant evidence supporting a role for TRIM22 as an anti-HIV-1 effector *in vivo.* They showed that expression of *TRIM22* in peripheral blood mononuclear cells (PBMCs) of HIV-1-infected individuals was significantly increased in patients after HIV-1 infection. Importantly, infected patients expressing higher *TRIM22* levels exhibited significantly lower viral loads and significantly higher CD4+ T-cell counts [26]. These findings are quite significant, as this suggests that TRIM22 has a potential effect on the severity and/or progression of HIV-1 infection. Additional research on the role of TRIM22 during primary infection will be important to provide a greater understanding of the effects TRIM22 may have on HIV-1 replication *in vivo*.

The antiviral activities of TRIM22 are not limited to HIV-1. In 2009, Eldin et al. identified TRIM22 as a potent inhibitor of encephalomyocarditis virus (EMCV) replication. TRIM22 was shown to interact with the EMCV 3C protease via the C-terminal domain of TRIM22, and expression of TRIM22 corresponded with increased ubiquitination of the 3C protease (Figure 3). 3C protease is essential for successful viral replication and has several roles, including processing of the viral polyprotein and inhibition of the host immune defences [11]. There are also reports that TRIM22 may play an important role in protecting the liver from viral pathogens. In 2009, Gao et al. reported that TRIM22 is highly upregulated in response to type I or II IFN in the hepatocellular carcinoma cell line HepG2. Cotransfection of plasmids encoding TRIM22 and replication-competent hepatitis B virus (HBV) inhibited the accumulation of HBV antigens in the supernatants of cells and significantly reduced levels of intracellular HBV RNA and DNA replication intermediates. Similar results were observed in the sera of mice during codelivery of plasmids to mouse livers, showing that TRIM22 can restrict HBV infection in an *in vivo* system. Using a luciferase reporter plasmid, they showed that TRIM22 downregulates expression from the HBV core promoter (Figure 3). This mechanism of action was dependent on the nuclear localization of TRIM22 and its E3 ubiquitin ligase activity [13]. Although there is no direct evidence for a protective role of TRIM22 against HBV in primates, *TRIM22* expression is significantly upregulated during clearance of HBV in chimpanzees [51]. Moreover, TRIM22 expression is significantly upregulated during clearance of hepatitis C virus (HCV) in chimpanzees [52]. These findings are paralleled in human infections, as *TRIM22* is significantly upregulated in cirrhotic liver from HCV patients and patients with mild chronic HCV infection and no fibrosis [53]. Further research is needed to assess the role of TRIM22 in inhibiting HBV and HCV *in vivo*.

In further support of the notion that TRIM22 is involved in the host antiviral response, TRIM22 expression is modulated in response to several other viruses and viral antigens (Table 2). TRIM22 expression is upregulated in response to infection with rubella virus [54] and Epstein-Barr virus (EBV) [55] and downregulated during infection with human papillomavirus type 31 [56]. A couple intriguing reports elude to the possibility that TRIM22 may also contribute to viral latency. Exogenous expression of TRIM22 significantly upregulates expression of the EBV latent membrane protein 1 (LMP-1) [55]. LMP-1 is required for latency during EBV infection and appears to induce an antiviral state by upregulating expression of several ISGs via an IFN- and STAT1-independent mechanism. The Kaposi's sarcoma-associated herpesvirus (KSHV) latency-associated nuclear antigen (LANA) also activates several ISGs including TRIM22, which was shown to be upregulated by LANA both in culture and in tissues from KSHV lesions. LANA also repressed transcription from the HIV-1 LTR, an NF*κ*B consensus sequence, and the SV40 promoter [57]. Furthermore, TRIM22 is expressed in resting T cells, which are known reservoirs of latent HIV-1, and is strongly repressed during T-cell activation [47]. Although much more research is needed to directly implicate TRIM22 in viral latency, it is tempting to hypothesize that TRIM22 contributes to viral latency.

### 4.3. Other Functions of TRIM22

Several reports in the literature suggest that TRIM22 may have a role in other biological processes, such as cell differentiation and proliferation. One group showed that the expression of TRIM22 is directly activated by p53 in myeloid cells via a functional p53-response element in intron 1 of the *TRIM22* gene [46]. They also showed that the p53-family member p73 can bind to this response element and activate *TRIM22 *expression [46]. Since p73 has been linked to the differentiation of leukemic cells [62], the authors speculated that TRIM22 may be involved in cell differentiation. Another group reported that TRIM22 expression is significantly upregulated during differentiation of the promyelocytic cell line NB4 [63]. They also showed that TRIM22 expression is high in monocytes and early granulocytes but decreases in the lymphocyte and late granulocyte populations and is undetectable in erythroid cells [63]. Obad et al. (2004) provided the first direct evidence supporting an antiproliferative role for TRIM22 by showing that overexpression of TRIM22 in the promonocytic cell line U937 resulted in decreased clonogenic growth [46]. An inverse correlation between TRIM22 expression and cell differentiation has also been reported, showing that TRIM22 is highly expressed in human immature CD34^+^ bone marrow progenitor cells, but declines in mature populations [63]. Despite the correlations of TRIM22 expression levels with cell differentiation and proliferation, the evidence lacks key experiments such as loss-of-function studies (i.e., TRIM22 knockdown) to conclusively implicate TRIM22 as a key player in any of these processes.

A couple of reports have associated TRIM22 with human disease. Downregulation of TRIM22 expression is associated with progression, relapse and increased mortality in cases of Wilms tumor [60, 61]. Although TRIM22 is a p53-responsive gene and may promote cell-cycle arrest [46], its role in tumour development and progression, including Wilms tumor, is yet to be determined. The involvement of TRIM proteins in cancer is not unprecedented. TRIM13, 24, and 29, which are also involved in p53 regulation, have also been implicated as important regulators for carcinogenesis. Moreover, TRIM19/PML may act as a tumour suppressor protein (reviewed in [4]). TRIM22 expression is also downregulated in CD4+ T cells from patients with active systemic lupus erythematosus (SLE) [59]. Although it is also unclear what role TRIM22 plays in this disease, it is notable that several other TRIM proteins, including TRIM 21, 25, 56, and 68, have been linked to SLE and other autoimmune diseases [5]. It will be interesting to learn more about the role (if any) TRIM22 plays in these and other human diseases.

Although it is clear that TRIM22 is an exciting and dynamic protein, it appears that we have only begun to understand its role in cellular biology and antiviral immunity. A rich evolutionary history, together with its potential involvement in numerous biological processes, suggests that TRIM22 is an important and multifarious protein. Despite its importance, the function of TRIM22 remains poorly understood, and a number of issues will need to be addressed in future research. One discrepancy that needs clarification is the disparate observations and contradictory reports surrounding TRIM22 subcellular localization. In particular, we need to understand why TRIM22 localization is so heterogeneous, as this may provide useful insight into its biological function. Another priority will be to consolidate previous reports on the antiviral mechanism of TRIM22. In the case of HIV-1, it will be important to determine the stage(s) of the virus lifecycle that TRIM22 targets. In this regard, future studies that identify the host and/or virus targets of TRIM22 will be extremely useful. In addition, it will be interesting to discover if TRIM22 has antiviral activity against additional viruses, and to determine the role it plays in other nonviral diseases. Overall, its breadth of involvement in antiviral immunity, combined with the range of possible mechanisms by which TRIM22 acts, presents a number of exciting research opportunities. Future work on TRIM22 will help us understand this important player in the host antiviral response and contribute to our knowledge of host-pathogen interactions.

## Figures and Tables

**Table 1:** Summary of the localization patterns observed for TRIM22.

Localization	Pattern	Cell Type	Epitope Tag	Reference
Cytoplasm	Diffuse Diffuse Diffuse Diffuse with speckles/bodies Diffuse Diffuse Diffuse with speckles/bodies	293T COS7 HeLa HeLa HeLa PBMCs U2OS	GFP or V5/His GFP or V5/His Endogenous GFP GFP or V5/His Endogenous GFP	[40] [40] [40] [22] [40] [40] [22]

Cytoplasm & Nucleus	Nucleoplasmic, with nuclear bodies^1^ Diffuse throughout, or nuclear bodies^2^ Nucleoplasmic, with nuclear bodies Diffuse, with cytoplasmic bodies^3^ Nucleoplasmic with NB^4^ Nucleoplasmic, with nuclear bodies Nucleoplasmic and cytoplasmic Diffuse with speckles^5-7^	ABC28 HeLa HeLa HeLa MCF7 MCF7 T47D U2OS	Endogenous EGFP Endogenous FLAG EGFP, EYFP, or FLAG Endogenous Endogenous Endogenous	[30] [30] [30] [43] [30] [30] [30] [42]

Nucleus	Aggregates/bodies Aggregates/bodies Diffuse with speckles/bodies Diffuse with speckles/bodies Diffuse with bodies	293 COS7 HepG2 HepG2 MCF7	Myc Myc Endogenous Myc FLAG	[41] [10] [13] [13] [20]

^1^Some colocalization with fibrillarin (Nucleoli).

^2^Pattern changes with cell cycle phase: (G0/G1: nuclear bodies; S-phase: nuclear speckles and cytoplasmic; mitosis: diffuse throughout cell).

^3^TRIM22 plasmid was coexpressed with Rhesus TRIM5*α*.

^4^Partial colocalization with Cajal bodies.

^5^Potential colocalization with calnexin (Endoplasmic reticulum).

^6^Localization was primarily cytoplasmic when cells were fixed with paraformaldehyde, or both cytoplasmic and nuclear when fixed with ice-cold methanol.

^7^Partial colocalization with the centrosome.

**Table 2:** Summary of factors that alter TRIM22 expression.

Stimulation	Change	Tissue	Reference
*Cytokines*			
IFN-*α*	increase	CEM, Jurkat, and THP-1 cells	[26]
IFN-*α*	increase	H9 cells	[47]
IFN-*α*	increase	HepG2 cells	[13]
IFN-*α*	increase	Primary MDM	[24]
IFN-*α*	increase	U937	[25]
IFN-*α*	increase	U-937-4 cells	[46]
IFN-*α*/*β*	increase	Daudi, and HeLa cells	[45]
IFN-*β*	increase	HOS cells	[14]
IFN-*γ*	increase	HeLa cells	[30, 45]
IFN-*γ*	increase	HepG2 cells	[13, 48, 49]
IFN-*γ*	increase	MCF7 cells	[30]
IL-1-*β*	increase	Coronary artery endothelium	[58]
IL-2	increase	CD4+, CD8+, NK cells	[50]
IL-15	increase	CD4+, CD8+, NK cells	[50]
Progesterone	increase	ABC28, and T47D cells	[30]
TNF-*α*	increase	Coronary artery endothelium	[58]

*Antigens/Infections*			
EBV infection^1^	increase	BL41-EBV cells^1^	[55]
EBV LMP-1	increase	DG75 cells	[55]
Hepatitis B virus infection^2^	increase	Liver tissue^2^	[51]
Hepatitis C virus infection^2^	increase	Liver tissue^2^	[52]
Hepatitis C virus infection	increase	Liver tissue	[53]
HIV-1 infection	increase	Immature DC	[55]
HIV-1 infection	increase	Primary MDM	[24]
HIV-1 infection	increase	Primary PBMCs	[26]
HIV-1 Tat	increase	Immature DC	[55]
HPV infection	decrease	Human keratinocytes	[56]
KSHV infection	increase	KSHV lesion	[57]
KSHV LANA	increase	BJAB cells	[57]
LPS	increase	Primary MDM	[24]
Rubella virus infection	increase	ECV304 cells	[54]

*Activation/Differentiation/Cell Cycle*			
1*α*,25-dihydroxyvitamine D3^3^	increase	Primary MDM	[24]
Anti-CD2	increase	Primary T cells	[47]
Anti-CD2/CD28	decrease	Primary T cells	[47]
Anti-CD2/CD28/CD3	decrease	CD4+, CD8+, NK cells^4^	[50]
All-trans retinoic acid	increase	HL60 and NB4 cells	[46]
All-trans retinoic acid	increase	Primary MDM	[24]
p53	increase	K562 and U-937-4 cells^5^	[46]
p73	increase	U-937-4 cells	[46]
Pioglitazone	increase	Primary MDM	[24]
UV-irradiation^6^	increase	MCF-7 cells	[46]

*Disease*			
SLE	decrease	CD4+ T cells from SLE patient	[59]
Wilms tumor	decrease	Tumor tissue	[60, 61]

^1^BL41 cells that are latently infected with EBV.

^2^From infected chimpanzees.

^3^Hormonally active form of Vitamin D.

^4^Only reached significance in CD8+ cells.

^5^Cells lack endogenous p53 but stably express a plasmid encoding p53 under control of a temperature-sensitive promoter. Cells were grown at the permissive temperature (32°C) to induce p53 expression.

^6^UV-irradiation induces p53 expression.
